# Methyl 3-[(1-butyl-1*H*-indol-3-yl)carbonyl­amino]propionate

**DOI:** 10.1107/S1600536809029973

**Published:** 2009-08-08

**Authors:** Gang Huang, Xing Yan Xu, Xiang Chao Zeng, Gui Hong Tang, Dong Dong Li

**Affiliations:** a Department of Chemistry, Jinan University, Guangzhou, Guangdong 510632, People’s Republic of China

## Abstract

In the title mol­ecule, C_17_H_22_N_2_O_3_, the mean plane of the terminal (C=O)OMe fragment and the indole plane form a dihedral angle of 78.94 (3)°. Inter­molecular N—H⋯O hydrogen bonds link the mol­ecules into chains extended along the *c* axis. The crystal packing exhibits π–π inter­actions, indicated by the short distance of 3.472 (2) Å between the centroids of the five-membered heterocycles of neighbouring mol­ecules.

## Related literature

For the bioactivity of indole derivatives, see: Fabio *et al.* (2007 ▶); Sharma *et al.* (2004 ▶). For related structures, see: Zeng *et al.* (2005 ▶); Siddiquee *et al.* (2009 ▶).
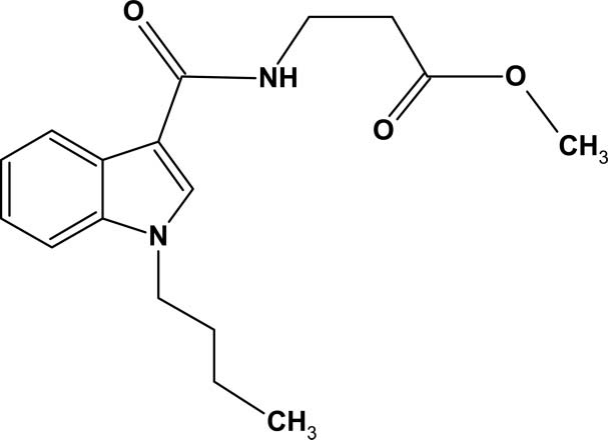


## Experimental

### 

#### Crystal data

C_17_H_22_N_2_O_3_
*M* *_r_* = 302.37
Monoclinic, 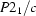

*a* = 14.144 (3) Å
*b* = 12.685 (3) Å
*c* = 9.198 (2) Å
β = 107.151 (4)°
*V* = 1576.8 (6) Å^3^
*Z* = 4
Mo *K*α radiation
μ = 0.09 mm^−1^
*T* = 173 K
0.46 × 0.42 × 0.17 mm

#### Data collection

Bruker SMART 1K CCD area-detector diffractometer
Absorption correction: multi-scan (*SADABS*; Sheldrick, 1996 ▶) *T*_min_ = 0.961, *T*_max_ = 0.985
7760 measured reflections
3093 independent reflections
2169 reflections with *I* > 2σ(*I*)
*R*_int_ = 0.037

#### Refinement

*R*[*F* ^2^ > 2σ(*F* ^2^)] = 0.045
*wR*(*F* ^2^) = 0.135
*S* = 1.05
3093 reflections
201 parameters
H-atom parameters constrained
Δρ_max_ = 0.23 e Å^−3^
Δρ_min_ = −0.27 e Å^−3^

### 

Data collection: *SMART* (Bruker,1999 ▶); cell refinement: *SAINT-Plus* (Bruker, 1999 ▶); data reduction: *SAINT-Plus*; program(s) used to solve structure: *SHELXS97* (Sheldrick, 2008 ▶); program(s) used to refine structure: *SHELXL97* (Sheldrick, 2008 ▶); molecular graphics: *SHELXTL* (Sheldrick, 2008 ▶); software used to prepare material for publication: *SHELXTL*.

## Supplementary Material

Crystal structure: contains datablocks I, global. DOI: 10.1107/S1600536809029973/cv2594sup1.cif

Structure factors: contains datablocks I. DOI: 10.1107/S1600536809029973/cv2594Isup2.hkl

Additional supplementary materials:  crystallographic information; 3D view; checkCIF report

## Figures and Tables

**Table 1:** Hydrogen-bond geometry (Å, °)

*D*—H⋯*A*	*D*—H	H⋯*A*	*D*⋯*A*	*D*—H⋯*A*
N2—H2⋯O1^i^	0.88	2.07	2.860 (2)	149

Symmetry code: (i) 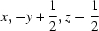.

**Fig. 1.**
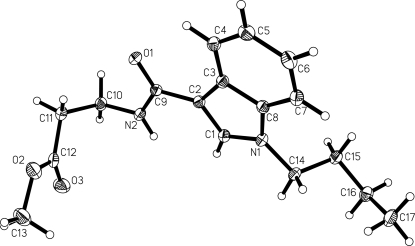
The molecular structure of the title compound, with the atom-numbering scheme. Displacement ellipsoids are drawn at the 30% probability level.

## supplementary crystallographic information

### Comment

Many indole derivatives show important bioactivities, such as metabotropic receptor antagonists (Fabio *et al.*, 2007) and protein kinase inhibiting activity (Sharma *et al.*, 2004). In continuation of our previous structural investigations of 3-trichloroacetylindole (Zeng *et al.*, 2005), we report here the crystal structure of the title compound, (I).

In (I) (Fig.1), all bond lengths and angles are unexceptional and correspond to those observed in the related compounds (Zeng *et al.*, 2005; Siddiquee *et al.*, 2009). In the crystal structure, adjacent molecules are linked through N2—H2A···O1 hydrogen bonds, forming chains extending along the *c* axis. The crystal packing exhibits π–π interactions proved by short distance of 3.472 (2) Å between the centroids of five-membered heterocycles of the neighbouring molecules.

### Experimental

A suspension of potassium carbonate (1.80 g, 13.0 mmol), 1-bromobutane (0.35 ml, 3.25 mmol) and methyl 3-(1*H*-Indole-3-carbonyl)aminopropionate (0.80 g, 3.25 mmol) in acetonitrile (30 ml) magnetically stirred at 328 K for 72 h. After filtration, the filtrate was evaporated *in vacuo*, and the residue was recrystallized with ethanol/water solution (1:1 *v*/*v*). Then the recrystallized solid was further purified by column chromatography on silica gel (petroleum ether/EtOAc, 1:1 *v*/*v*) to yield I (m.p. 367 K, 91.6%). Colourless crystals suitable for X-ray analysis were obtained over a period of five days by slow evaporation at room temperature of a petroleum ether/EtOAc solution (1:1 *v*/*v*).

### Refinement

The H atoms were positioned geometrically [C—H = 0.99Å for CH_2_, 0.98Å for CH_3_, 0.95Å for CH(aromatic) and N—H = 0.88 Å] and refined using a riding model, with *U*_iso_ = 1.2*U*_eq_ (1.5*U*_eq_ for the methyl group) of the parent atom.

### Crystal data

**Table:**

C_17_H_22_N_2_O_3_	*D*_x_ = 1.274 Mg m^−^^3^
*M**_r_* = 302.37	Melting point: 367 K
Monoclinic, *P*2_1_/*c*	Mo *K*α radiation, λ = 0.71073 Å
*a* = 14.144 (3) Å	Cell parameters from 2799 reflections
*b* = 12.685 (3) Å	θ = 2.8–26.9°
*c* = 9.198 (2) Å	µ = 0.09 mm^−^^1^
β = 107.151 (4)°	*T* = 173 K
*V* = 1576.8 (6) Å^3^	Plate, colourless
*Z* = 4	0.46 × 0.42 × 0.17 mm
*F*(000) = 648	

### Data collection

**Table:**

Bruker SMART 1K CCD area-detector diffractometer	3093 independent reflections
Radiation source: fine-focus sealed tube	2169 reflections with *I* > 2σ(*I*)
graphite	*R*_int_ = 0.037
φ and ω scans	θ_max_ = 26.0°, θ_min_ = 2.2°
Absorption correction: multi-scan (*SADABS*; Sheldrick, 1996)	*h* = −17→9
*T*_min_ = 0.961, *T*_max_ = 0.985	*k* = −15→13
7760 measured reflections	*l* = −11→10

### Refinement

**Table:**

Refinement on *F*^2^	Primary atom site location: structure-invariant direct methods
Least-squares matrix: full	Secondary atom site location: difference Fourier map
*R*[*F*^2^ > 2σ(*F*^2^)] = 0.045	Hydrogen site location: inferred from neighbouring sites
*wR*(*F*^2^) = 0.135	H-atom parameters constrained
*S* = 1.05	*w* = 1/[σ^2^(*F*_o_^2^) + (0.0677*P*)^2^ + 0.3821*P*] where *P* = (*F*_o_^2^ + 2*F*_c_^2^)/3
3093 reflections	(Δ/σ)_max_ = 0.001
201 parameters	Δρ_max_ = 0.23 e Å^−^^3^
0 restraints	Δρ_min_ = −0.27 e Å^−^^3^

### Special details

**Table:**

Geometry. All e.s.d.'s (except the e.s.d. in the dihedral angle between two l.s. planes) are estimated using the full covariance matrix. The cell e.s.d.'s are taken into account individually in the estimation of e.s.d.'s in distances, angles and torsion angles; correlations between e.s.d.'s in cell parameters are only used when they are defined by crystal symmetry. An approximate (isotropic) treatment of cell e.s.d.'s is used for estimating e.s.d.'s involving l.s. planes.
Refinement. Refinement of *F*^2^ against ALL reflections. The weighted *R*-factor *wR* and goodness of fit *S* are based on *F*^2^, conventional *R*-factors *R* are based on *F*, with *F* set to zero for negative *F*^2^. The threshold expression of *F*^2^ > σ(*F*^2^) is used only for calculating *R*-factors(gt) *etc*. and is not relevant to the choice of reflections for refinement. *R*-factors based on *F*^2^ are statistically about twice as large as those based on *F*, and *R*- factors based on ALL data will be even larger.

### Fractional atomic coordinates and isotropic or equivalent isotropic displacement parameters (Å^2^)

**Table:**

	*x*	*y*	*z*	*U*_iso_*/*U*_eq_	
O1	0.32207 (9)	0.17769 (11)	1.18054 (15)	0.0326 (3)	
N2	0.27815 (11)	0.22657 (13)	0.93470 (18)	0.0286 (4)	
H2	0.2944	0.2301	0.8495	0.034*	
N1	0.56995 (11)	0.12769 (12)	0.95991 (17)	0.0271 (4)	
O2	0.07347 (10)	0.00166 (12)	0.83159 (18)	0.0431 (4)	
C1	0.47775 (13)	0.16910 (14)	0.9284 (2)	0.0255 (4)	
H1	0.4419	0.1987	0.8331	0.031*	
C2	0.44315 (13)	0.16250 (14)	1.0527 (2)	0.0251 (4)	
O3	0.06776 (13)	0.13614 (14)	0.67382 (18)	0.0545 (5)	
C3	0.52016 (13)	0.11227 (14)	1.1707 (2)	0.0259 (4)	
C9	0.34451 (13)	0.19061 (15)	1.0605 (2)	0.0258 (4)	
C4	0.53054 (14)	0.07969 (15)	1.3199 (2)	0.0300 (4)	
H4	0.4797	0.0931	1.3659	0.036*	
C12	0.08303 (14)	0.10345 (18)	0.8007 (2)	0.0343 (5)	
C14	0.62646 (14)	0.11611 (16)	0.8515 (2)	0.0292 (4)	
H14A	0.5822	0.1302	0.7479	0.035*	
H14B	0.6494	0.0422	0.8539	0.035*	
C7	0.68374 (14)	0.03955 (15)	1.1879 (2)	0.0312 (5)	
H7	0.7354	0.0266	1.1436	0.037*	
C8	0.59785 (13)	0.09149 (14)	1.1080 (2)	0.0255 (4)	
C11	0.11220 (14)	0.16838 (17)	0.9426 (2)	0.0328 (5)	
H11A	0.1460	0.1225	1.0294	0.039*	
H11B	0.0517	0.1965	0.9619	0.039*	
C10	0.18006 (13)	0.25988 (16)	0.9350 (2)	0.0310 (5)	
H10A	0.1496	0.3012	0.8416	0.037*	
H10B	0.1862	0.3068	1.0234	0.037*	
C15	0.71526 (14)	0.18848 (16)	0.8815 (2)	0.0300 (5)	
H15A	0.7627	0.1708	0.9813	0.036*	
H15B	0.6936	0.2623	0.8862	0.036*	
C16	0.76656 (15)	0.17862 (17)	0.7580 (2)	0.0364 (5)	
H16A	0.7164	0.1870	0.6576	0.044*	
H16B	0.8146	0.2371	0.7698	0.044*	
C5	0.61526 (15)	0.02801 (16)	1.3987 (2)	0.0345 (5)	
H5	0.6227	0.0055	1.4999	0.041*	
C6	0.69128 (15)	0.00763 (16)	1.3333 (2)	0.0353 (5)	
H6	0.7489	−0.0288	1.3906	0.042*	
C17	0.82035 (16)	0.07542 (19)	0.7591 (3)	0.0455 (6)	
H17A	0.8722	0.0676	0.8564	0.068*	
H17B	0.8504	0.0749	0.6757	0.068*	
H17C	0.7734	0.0169	0.7461	0.068*	
C13	0.04133 (18)	−0.0678 (2)	0.7031 (3)	0.0564 (7)	
H13A	−0.0213	−0.0421	0.6344	0.085*	
H13B	0.0319	−0.1389	0.7382	0.085*	
H13C	0.0915	−0.0698	0.6489	0.085*	

### Atomic displacement parameters (Å^2^)

**Table:**

	*U*^11^	*U*^22^	*U*^33^	*U*^12^	*U*^13^	*U*^23^
O1	0.0298 (7)	0.0442 (9)	0.0277 (7)	−0.0011 (6)	0.0144 (6)	−0.0037 (6)
N2	0.0250 (8)	0.0355 (9)	0.0278 (9)	0.0011 (7)	0.0117 (7)	−0.0009 (7)
N1	0.0256 (8)	0.0307 (9)	0.0281 (9)	0.0003 (7)	0.0124 (7)	−0.0005 (7)
O2	0.0347 (8)	0.0382 (9)	0.0536 (10)	−0.0014 (7)	0.0088 (7)	−0.0073 (7)
C1	0.0233 (9)	0.0261 (10)	0.0274 (10)	0.0004 (8)	0.0078 (8)	0.0002 (8)
C2	0.0251 (9)	0.0247 (10)	0.0266 (10)	−0.0027 (8)	0.0095 (8)	−0.0022 (8)
O3	0.0636 (11)	0.0652 (12)	0.0348 (9)	−0.0229 (9)	0.0148 (8)	−0.0042 (8)
C3	0.0255 (10)	0.0233 (9)	0.0301 (10)	−0.0034 (8)	0.0100 (8)	−0.0026 (8)
C9	0.0269 (10)	0.0252 (10)	0.0272 (10)	−0.0047 (8)	0.0107 (8)	−0.0061 (8)
C4	0.0307 (10)	0.0299 (10)	0.0312 (10)	−0.0062 (8)	0.0122 (8)	−0.0014 (8)
C12	0.0216 (10)	0.0450 (13)	0.0387 (12)	−0.0051 (9)	0.0128 (9)	−0.0032 (10)
C14	0.0282 (10)	0.0329 (11)	0.0307 (11)	0.0002 (8)	0.0152 (8)	−0.0041 (8)
C7	0.0273 (10)	0.0287 (10)	0.0385 (12)	0.0001 (8)	0.0109 (9)	−0.0005 (9)
C8	0.0254 (10)	0.0221 (9)	0.0297 (10)	−0.0039 (7)	0.0089 (8)	−0.0034 (8)
C11	0.0255 (10)	0.0413 (12)	0.0341 (11)	−0.0018 (9)	0.0129 (8)	−0.0013 (9)
C10	0.0246 (10)	0.0323 (11)	0.0377 (11)	0.0024 (8)	0.0116 (8)	−0.0032 (9)
C15	0.0303 (10)	0.0304 (10)	0.0333 (11)	−0.0004 (8)	0.0158 (8)	−0.0008 (8)
C16	0.0354 (11)	0.0419 (12)	0.0371 (12)	−0.0047 (9)	0.0187 (9)	0.0014 (9)
C5	0.0388 (12)	0.0337 (11)	0.0293 (11)	−0.0044 (9)	0.0074 (9)	0.0054 (9)
C6	0.0321 (11)	0.0300 (11)	0.0401 (12)	0.0014 (9)	0.0049 (9)	0.0055 (9)
C17	0.0348 (12)	0.0590 (16)	0.0483 (14)	0.0078 (11)	0.0211 (11)	−0.0015 (11)
C13	0.0407 (13)	0.0510 (15)	0.0742 (18)	−0.0051 (11)	0.0118 (12)	−0.0255 (13)

### Geometric parameters (Å, °)

**Table:**

O1—C9	1.247 (2)	C7—C8	1.387 (3)
N2—C9	1.337 (2)	C7—H7	0.9500
N2—C10	1.451 (2)	C11—C10	1.521 (3)
N2—H2	0.8800	C11—H11A	0.9900
N1—C1	1.356 (2)	C11—H11B	0.9900
N1—C8	1.380 (2)	C10—H10A	0.9900
N1—C14	1.458 (2)	C10—H10B	0.9900
O2—C12	1.337 (3)	C15—C16	1.523 (3)
O2—C13	1.436 (3)	C15—H15A	0.9900
C1—C2	1.373 (3)	C15—H15B	0.9900
C1—H1	0.9500	C16—C17	1.513 (3)
C2—C3	1.440 (3)	C16—H16A	0.9900
C2—C9	1.462 (3)	C16—H16B	0.9900
O3—C12	1.197 (2)	C5—C6	1.402 (3)
C3—C4	1.399 (3)	C5—H5	0.9500
C3—C8	1.408 (3)	C6—H6	0.9500
C4—C5	1.371 (3)	C17—H17A	0.9800
C4—H4	0.9500	C17—H17B	0.9800
C12—C11	1.495 (3)	C17—H17C	0.9800
C14—C15	1.514 (3)	C13—H13A	0.9800
C14—H14A	0.9900	C13—H13B	0.9800
C14—H14B	0.9900	C13—H13C	0.9800
C7—C6	1.371 (3)		
			
C9—N2—C10	121.69 (16)	C12—C11—H11B	108.9
C9—N2—H2	119.2	C10—C11—H11B	108.9
C10—N2—H2	119.2	H11A—C11—H11B	107.7
C1—N1—C8	108.50 (15)	N2—C10—C11	113.24 (16)
C1—N1—C14	125.40 (16)	N2—C10—H10A	108.9
C8—N1—C14	125.93 (16)	C11—C10—H10A	108.9
C12—O2—C13	116.39 (19)	N2—C10—H10B	108.9
N1—C1—C2	110.77 (16)	C11—C10—H10B	108.9
N1—C1—H1	124.6	H10A—C10—H10B	107.7
C2—C1—H1	124.6	C14—C15—C16	111.46 (16)
C1—C2—C3	106.16 (16)	C14—C15—H15A	109.3
C1—C2—C9	127.25 (17)	C16—C15—H15A	109.3
C3—C2—C9	126.32 (16)	C14—C15—H15B	109.3
C4—C3—C8	118.55 (17)	C16—C15—H15B	109.3
C4—C3—C2	135.00 (17)	H15A—C15—H15B	108.0
C8—C3—C2	106.41 (16)	C17—C16—C15	114.53 (17)
O1—C9—N2	120.95 (17)	C17—C16—H16A	108.6
O1—C9—C2	120.46 (17)	C15—C16—H16A	108.6
N2—C9—C2	118.54 (16)	C17—C16—H16B	108.6
C5—C4—C3	118.83 (18)	C15—C16—H16B	108.6
C5—C4—H4	120.6	H16A—C16—H16B	107.6
C3—C4—H4	120.6	C4—C5—C6	121.53 (19)
O3—C12—O2	122.8 (2)	C4—C5—H5	119.2
O3—C12—C11	125.8 (2)	C6—C5—H5	119.2
O2—C12—C11	111.45 (18)	C7—C6—C5	121.00 (18)
N1—C14—C15	113.98 (15)	C7—C6—H6	119.5
N1—C14—H14A	108.8	C5—C6—H6	119.5
C15—C14—H14A	108.8	C16—C17—H17A	109.5
N1—C14—H14B	108.8	C16—C17—H17B	109.5
C15—C14—H14B	108.8	H17A—C17—H17B	109.5
H14A—C14—H14B	107.7	C16—C17—H17C	109.5
C6—C7—C8	117.49 (18)	H17A—C17—H17C	109.5
C6—C7—H7	121.3	H17B—C17—H17C	109.5
C8—C7—H7	121.3	O2—C13—H13A	109.5
N1—C8—C7	129.22 (17)	O2—C13—H13B	109.5
N1—C8—C3	108.14 (16)	H13A—C13—H13B	109.5
C7—C8—C3	122.60 (18)	O2—C13—H13C	109.5
C12—C11—C10	113.33 (16)	H13A—C13—H13C	109.5
C12—C11—H11A	108.9	H13B—C13—H13C	109.5
C10—C11—H11A	108.9		
			
C8—N1—C1—C2	0.6 (2)	C1—N1—C8—C7	177.15 (19)
C14—N1—C1—C2	176.18 (16)	C14—N1—C8—C7	1.6 (3)
N1—C1—C2—C3	−0.4 (2)	C1—N1—C8—C3	−0.6 (2)
N1—C1—C2—C9	−174.69 (17)	C14—N1—C8—C3	−176.13 (16)
C1—C2—C3—C4	−177.5 (2)	C6—C7—C8—N1	−177.13 (18)
C9—C2—C3—C4	−3.0 (3)	C6—C7—C8—C3	0.3 (3)
C1—C2—C3—C8	0.0 (2)	C4—C3—C8—N1	178.29 (16)
C9—C2—C3—C8	174.42 (17)	C2—C3—C8—N1	0.3 (2)
C10—N2—C9—O1	4.9 (3)	C4—C3—C8—C7	0.4 (3)
C10—N2—C9—C2	−177.58 (16)	C2—C3—C8—C7	−177.55 (17)
C1—C2—C9—O1	177.91 (18)	O3—C12—C11—C10	−35.6 (3)
C3—C2—C9—O1	4.7 (3)	O2—C12—C11—C10	145.76 (17)
C1—C2—C9—N2	0.4 (3)	C9—N2—C10—C11	−74.0 (2)
C3—C2—C9—N2	−172.86 (17)	C12—C11—C10—N2	−69.1 (2)
C8—C3—C4—C5	−0.6 (3)	N1—C14—C15—C16	−175.70 (16)
C2—C3—C4—C5	176.6 (2)	C14—C15—C16—C17	−70.2 (2)
C13—O2—C12—O3	−1.3 (3)	C3—C4—C5—C6	0.2 (3)
C13—O2—C12—C11	177.36 (17)	C8—C7—C6—C5	−0.7 (3)
C1—N1—C14—C15	110.6 (2)	C4—C5—C6—C7	0.5 (3)
C8—N1—C14—C15	−74.5 (2)		

### Hydrogen-bond geometry (Å, °)

**Table:**

*D*—H···*A*	*D*—H	H···*A*	*D*···*A*	*D*—H···*A*
N2—H2···O1^i^	0.88	2.07	2.860 (2)	149

Symmetry codes: (i) *x*, −*y*+1/2, *z*−1/2.
